# Effects of Acute Heat and Cold Exposures at Rest or during Exercise on Subsequent Energy Intake: A Systematic Review and Meta-Analysis

**DOI:** 10.3390/nu13103424

**Published:** 2021-09-28

**Authors:** Juliette Millet, Julien Siracusa, Pierre-Emmanuel Tardo-Dino, David Thivel, Nathalie Koulmann, Alexandra Malgoyre, Keyne Charlot

**Affiliations:** 1 Département Environnements Opérationnels, Institut de Recherche Biomédicale des Armées, Unité de Physiologie des Exercices et Activités en Conditions Extrêmes, 91223 Bretigny-Sur-Orge, France; millet.jlte@gmail.com (J.M.); siracusa.julien@gmail.com (J.S.); pierre-emmanuel.tardo-dino@intradef.gouv.fr (P.-E.T.-D.); nathalie1.koulmann@intradef.gouv.fr (N.K.); alexandra.malgoyre@intradef.gouv.fr (A.M.); 2 LBEPS, Univ Evry, IRBA, Université Paris Saclay, 91025 Evry, France; 3 Laboratory AME2P, University of Clermont Auvergne, 63170 Aubière, France; david.thivel@uca.fr; 4 Ecole du Val-de-Grâce, 1, Place Alphonse Laveran, 75230 Paris, France

**Keywords:** energy intake, energy expenditure, energy balance, hot ambient temperature, cold ambient temperature, exercise

## Abstract

The objective of this meta-analysis was to assess the effect of acute heat/cold exposure on subsequent energy intake (EI) in adults. We searched the following sources for publications on this topic: PubMed, Ovid Medline, Science Direct and SPORTDiscus. The eligibility criteria for study selection were: randomized controlled trials performed in adults (169 men and 30 women; 20–52 years old) comparing EI at one or more meals taken *ad libitum*, during and/or after exposure to heat/cold and thermoneutral conditions. One of several exercise sessions could be realized before or during thermal exposures. Two of the thirteen studies included examined the effect of heat (one during exercise and one during exercise and at rest), eight investigated the effect of cold (six during exercise and two at rest), and three the effect of both heat and cold (two during exercise and one at rest). The meta-analysis revealed a small increase in EI in cold conditions (g = 0.44; *p* = 0.019) and a small decrease in hot conditions (g = −0.39, *p* = 0.022) for exposure during both rest and exercise. Exposures to heat and cold altered EI in opposite ways, with heat decreasing EI and cold increasing it. The effect of exercise remains unclear.

## 1. Introduction

Athletes and military personnel are often required to go abroad for training, competition, or missions. During such travel, they may be exposed to harsh thermal conditions [1,2,3] that may alter their food intake with respect to a thermoneutral environment [4,5,6] and amplify the energy deficits that these populations already experience regularly even in the absence of thermal stress [7,8,9,10]. A prolonged negative energy balance may have critical effects on physical [11] and cognitive [12] performances, mood [13], and several health markers [14,15]. The identification of these potential risks during exposure to hot and cold environments, and the determination of their magnitudes is, therefore, of considerable importance in these specific populations.

There has been relatively little research on this topic, and most of the studies that have been conducted have focused on acute effects. However, the results of several studies seem to indicate opposite effects of heat and cold, regardless of whether exposure occurs during exercise or at rest. Indeed, subjects tend to eat more at lower temperatures [16,17,18] and to eat less at higher temperatures [18,19,20], regardless of whether the exposure to extreme temperatures occurs during intense physical activity or at rest. However, a large proportion of published studies reported no significant effect of exposure to heat or cold on appetitive responses [21,22,23,24,25,26].

It remains unclear whether exposures to heat or cold have an impact on energy intake. A first mini-review highlighted the heterogeneity of study methodology (type, intensity, and duration of exercise, environmental conditions, the nature of test meals, and sample characteristics), partly explaining the difficulty reaching a consensus [4]. However, the mini-review concerned did not consider exposures during a rest period [23,27] and several articles exploring the effect of temperature on energy intake have since been published [20,21,28,29].

The increasing number of publications on this topic and the growing need to provide recommendations for nutrition during exposure to extreme environments [1,30] have highlighted a need to determine the systematic impact of these environments on *ad libitum* energy intake. In this meta-analysis, we, therefore, aimed to assess the acute effects of exposure to heat or cold during rest and exercise on energy intake through an analysis of randomized control trials.

## 2. Methods

This systematic review was performed in accordance with the 2009 PRISMA statement [31]. The review protocol was registered with PROSPERO in March 2021 (registration number CRD42021247385).

### 2.1. Search Strategy

We searched PubMed, Ovid Medline, Science Direct and SPORTDiscus, without temporal restrictions, for relevant articles. The last literature search was performed on 31 May 2021. The search was performed with no restriction of publication date or type. The reference lists of recent publications on this topic and the studies cited were screened for additional references missed in the initial search. We searched for the following indexed terms in the title or abstract: (“Energy Intake”) AND (Heat OR Temperature OR Cold) AND (Physical Activity OR Exercise OR Rest).

### 2.2. Inclusion and Exclusion Criteria

We considered original articles and published conference abstracts. Book chapters, reviews and meta-analyses were automatically excluded. Only articles published in English were included.

Randomized controlled trials in healthy adults, comparing energy intake between individuals exposed to heat or cold and individuals remaining in thermoneutral conditions, were included. The thermoneutral zone is defined as conditions in which “temperature regulation is achieved only by control of sensible (dry) heat loss, i.e., without regulatory changes in metabolic heat production or evaporative heat loss” [32]. The thermoneutral zone lies between 27 and 34 °C in naked individuals, but its limits may be up to 10 °C lower in clothed individuals [33]. As clothing conditions differed between studies, we considered a range of 18–26 °C to constitute the thermoneutral zone. In water, we considered the thermoneutral zone to correspond to that without clothing in air. The temperature during exposure to heat or cold had to be at least 6 °C higher/lower than the limits of the thermoneutral zone to ensure an impact of the thermal exposure. Energy intake had to be measured by the gold standard procedure (weighing of the food and drink consumed), during one or several meals taken ad libitum. No restriction was applied to the temperature of the foods and drinks served (cold, hot, or at room temperature). We excluded studies that did not present mean absolute energy intake with the standard deviation (SD) or standard error of the mean (SEM). Data could be presented in textual (in the body of the manuscript or in tabular form) or graphical form. We contacted the authors of studies whose results were not published according to these standards, to obtain standardized results. Studies on children, adolescents (<18 years old), and the elderly (>65 years old) or in populations with diseases or eating disorders were excluded. Sex, training level and body composition (by fat mass or body mass index) were not used as inclusion or exclusion criteria. The duration of thermal exposure and its nature (aquatic or aerial, partial or full body) were not restricted. Thus, meals could be consumed either during or after the thermal exposure. No restriction was applied to the type of physical exercise (nature, duration, intensity), provided that the same activity was performed in both sessions.

### 2.3. Study Selection

One of the authors (J.M.) performed the search and another (K.C.) was responsible for adjudication. These two authors independently screened all titles and abstracts and retrieved the full text of any article considered definitely or possibly eligible. Both authors then reviewed the full text of the articles and compared them with the eligibility criteria. Any disagreement between the two authors was resolved by discussion with a third author (J.S.).

### 2.4. Data Extraction

Data on the characteristics of the included studies were extracted independently by J.M. and K.C.: (1) characteristics of the population (number of participants, age, height, weight, and body mass index); (2) characteristics of exposure (temperature, humidity, duration); (3) presence of physical activity (yes or no) and its characteristics (nature, ergometer measurements, duration, intensity); (4) characteristics of ad libitum meals (description of foods, number and timing of meals, temperature of the foods and drinks served); (5) description of clothing; (6) markers of perceptive and physiological thermal stress and the absolute difference between the heat/cold exposure and thermoneutral sessions; (7) mean energy intake (±SD or SEM).

Energy intake in kcal was converted to kJ (1 kcal = 4.1868 kJ) and SEM was converted into SD.

### 2.5. Assessment of the Risk of Bias

J.M. and K.C. independently assessed the risk of bias of each of the trials included. Any disagreement was discussed between the two authors, and a third author (J.S.) gave a final judgement if no consensus could be reached. Trials were assessed with the Cochrane tool for assessing risk of bias in randomized trials [34]. This tool evaluates the following domains: random sequence generation, allocation concealment, blinding of participants and staff, blinding to outcome assessment, incomplete outcome data, selective reporting, and other sources of bias. Each area was classified as being at low risk, unclear risk, or high risk of bias. The overall risk of bias was considered low if all domains had a low risk of bias, high if at least one domain had a high risk of bias, or unclear if at least one domain had an unclear risk of bias and no domain had a high risk. This rule is specified by the Cochrane tool for assessing risk of bias in randomized controlled trials, as any source of bias in a trial is problematic and there is little empirical research to favor one domain over another [34].

### 2.6. Meta-Analysis Procedures and Statistical Approach

The extracted data were entered into statistical software (Jamovi, Version 1.6, Sydney, Australia). The input data included sample size, the means and SD of energy intake under control and experimental conditions (heat or cold exposure).

The software calculated the standardized difference in means with a 95% confidence interval (CI) level to determine Hedge’s g, which was preferred over Cohen’s d as it is more suitable for small samples. As proposed by Cohen [35], these coefficients were used to interpret the size of any effect on energy intake of the experimental conditions (heat or cold) relative to control conditions (thermoneutral). Effect size (ES) was interpreted as follows: <0.2 was considered trivial, 0.2–0.3 small, 0.5–0.8 moderate, and >0.8 large [35]. A negative effect size indicated that thermal exposure decreased energy intake, whereas a positive effect size indicated that thermal exposure increased energy intake. Overall, effect sizes were calculated with a random-effects model accounting for true variation in effects between studies, together with random error within a single study. A random-effects model was preferred over a fixed-effects model because experimental factors, such as temperature, food type, and type of exercise, varied considerably and a random-effects model can account for such variations more accurately during analysis.

Heterogeneity was evaluated by calculating the I^2^ index and Cochrane’s Q. Values of 25%, 50%, and 75%, corresponding to low, moderate, and high heterogeneity, respectively, were used for the I^2^ analysis of heterogeneity [36]. Credible or prediction intervals are of recognized utility for understanding the heterogeneity of true effect sizes in meta-analyses. Cochrane’s Q was calculated to determine whether variations in the observed effect were likely to be due exclusively to sampling error. Significant sample heterogeneity exists if the Q value exceeds the degrees of freedom (df) of the estimate [37]. Sensitivity analyses were conducted by excluding one study at a time, to determine whether the overall results were influenced by a particular study. The results of the meta-analysis were visualized with a forest plot illustrating the results of the individual studies and the overall effect.

Funnel plots were used to identify publication bias. Egger’s regression test was used to quantify this asymmetry. In the absence of publication bias, studies should be uniformly distributed around the mean of the standardized differences due to sampling errors. An Egger’s *p*-value > 0.05 suggests that there is no evidence of publication bias.

## 3. Results

### 3.1. Search Results

**Figure 1:**
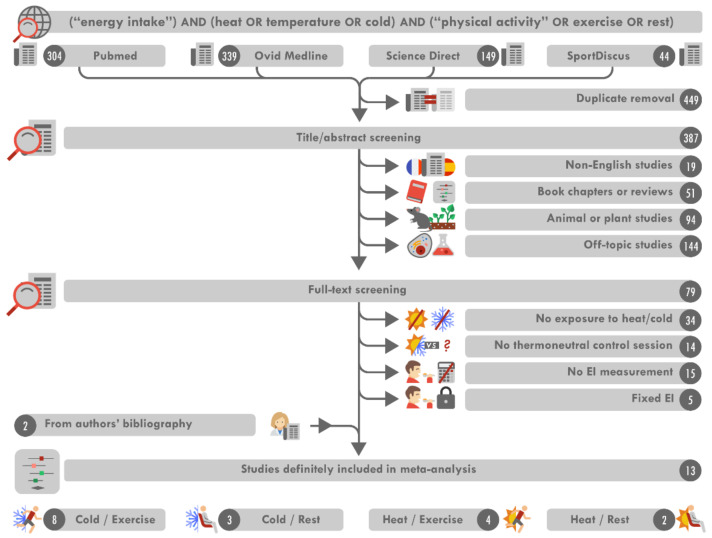
Flowchart for study inclusion. EI: energy intake.

A flowchart for study selection is shown in Figure 1. The search of the four databases identified 836 results. After eliminating duplicates, 387 articles matching the keywords remained. We excluded 270 of these articles after reviewing titles and abstracts, as these studies did not meet the selection criteria. We then excluded 68 of the remaining 79 articles after full-text review. Two studies [23,38] were then added after an analysis of the reference lists of the selected articles. Thus, in total, 13 studies were identified as meeting the inclusion criteria and were included in the analysis [16,17,18,19,20,21,22,23,27,28,29,38,39].

### 3.2. Study and Population Characteristics

**Figure 2:**
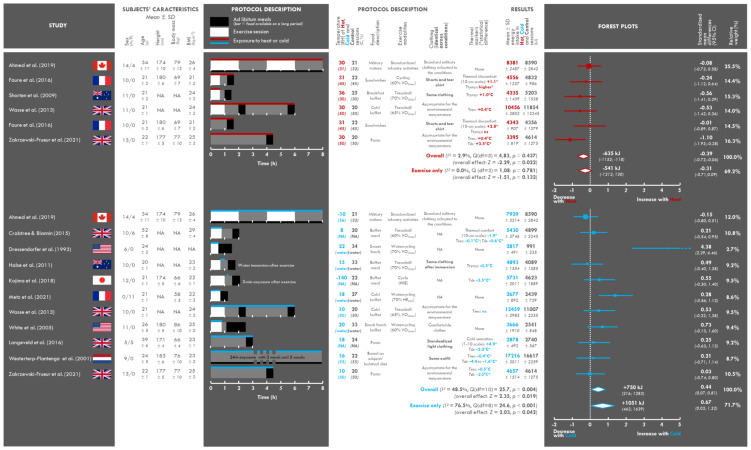
General characteristics of the included studies and random-effects model-based meta-analysis of energy intake during or after exposure to heat or cold relative to thermoneutral conditions. BMI: body mass index, RH: relative humidity, VO_2max_: maximal oxygen uptake, HRmax: maximal heart rate, Trec: rectal temperature, Ttymp: tympanic temperature, Tsk: skin temperature, CI: confidence interval.

The characteristics of the 13 studies included are presented in Figure 2. All the studies were laboratory experiments, published between 1993 and 2021, from seven different countries. The mean age of the participants in the studies was 27.3 years (*n* = 199, range: 20–52 years) and the mean BMI was 24.1 (*n* = 193, range: 21.3–28.9 kg.m^−2^). BMI was not reported in some studies [16,38], but was calculated here if the height and weight of the participants were reported. This was not, however, possible in one study [38]. All participants were young, lean, and active, with the exception of the study by Crabtree and Blannin [17], which included overweight and/or obese participants. Only one study was conducted in an exclusively female population [29] and nine were conducted in exclusively male populations [16,18,19,20,22,27,28,38,39]. The remaining three studies [17,21,23] included both male and female participants. Note that military participants were included in the study by Ahmed et al. [21].

### 3.3. Assessment of the Intervention: Temperature

The details of exposure to the different thermal environments are presented in Figure 2. Heat and cold exposure effects were assessed in six and eleven studies, respectively.

Mean temperature and relative humidity in the hot conditions were 31 ± 2 °C (range: 30–36 °C) and 42 ± 9% (30–50 %), respectively (vs. 22 ± 2 °C (20–25 °C) and 42 ± 9% (30–50%) in the thermoneutral control conditions). In one study, participants were exposed to heat only during the 40 min period of physical activity [19], whereas this exposure was maintained throughout the experiment in the other studies (exposure duration between 1 h 40 min and 8 h).

Cold exposure was either aerial (*n* = 7) or aquatic (*n* = 4). In studies with aerial exposure, the mean temperature was 9 ± 10 °C (−10–18 °C) (vs. 21 ± 2 °C (20–24 °C) in thermoneutral control conditions), other than in the study by Kojima et al. [28], in which cold was administered during a three minute cryogenic session at −140 °C. Relative humidity was almost never specified. In studies with immersion, mean water temperature was 19 ± 3 °C (15–22 °C) (vs. 32 ± 3 °C (27–34 °C) for thermoneutral control conditions). Two studies used a short post-exercise exposure to cold: 15 min in water at 15 °C (vs. 33 °C in the control session [39]) and a three-minute cryogenic session [28]. In five studies, participants were exposed to cold throughout the experiment (exposure duration between 3 and 8 h) [18,20,21,23,27], whereas cold exposure was maintained only during physical activities (30–45 min) in four studies [16,17,29,38].

### 3.4. Clothing Characteristics

Clothing was not systematically described and detail was often lacking. In studies of heat exposure, half the studies used similar standardized clothing in all conditions [19,22], whereas the others adapted the clothing to the environmental temperature, with either the imposition of military clothing [21] or a free choice of clothing by the participant [18,20]. In studies of cold exposure, clothing was not specified in four studies [17,28,29,38] and was insufficiently detailed in another [16]. Clothing was similar in three studies [23,27,39] and adapted to the thermal exposure in another three [18,20,21].

### 3.5. Assessment of the Perceptive and Physiological Impact of Exposure to Heat or Cold

Skin (in six of the seventeen sets of conditions) and core (rectal and tympanic; in nine among seventeen) temperatures were used to assess the physiological effect of heat/cold exposure, and thermal sensation and comfort scales (in four of the seventeen sets of conditions) were used to assess the perceptive effect of these exposures. During heat exposure, tympanic and rectal temperatures were almost always higher in individuals exposed to heat than in thermoneutral conditions at the start of the test meals [18,19,20,22], but this difference was significant in only one study [22]. Skin temperature was assessed in one study, which reported higher values (+3.5 °C) at the beginning of the meal in individuals exposed to heat [20]. Thermal discomfort at the start of the meal was found to be significantly higher in one study testing two sets of conditions [22]. During cold exposure, rectal [17,18,20,27], tympanic [39], and skin temperature [17,20,23,27,28] were lower in all studies at the start of the test meals; these decreases were significant in only a few studies for core temperature [17,27], but were always significant for skin temperature. Thermal comfort and cold sensation were significantly lower [17] and higher [23], respectively, in individuals exposed to cold.

### 3.6. Characteristics of the Food and Exercise Interventions

The details of the ad libitum meals and of the intervention are presented in Figure 2. All test meals were taken ad libitum. A single meal was presented to subjects at the end of exercise or rest in 12 experimental sessions. Two meals were presented in the middle and at the end of the sessions in one study, corresponding to two sessions [18]. Finally, two studies (each involving three sessions) presented food throughout the experiment [21,27]. Depending on the session, the meals were eaten either in the thermal environment tested (hot or cold) (*n* = 10) or in a thermoneutral environment (*n* = 7). The nature of the meals varied considerably and was not systematically described: foods and drinks served cold, hot, or at room temperature, buffet or single course (pasta or sandwich), usual food or items from military rations.

Physical activity was included in the protocol of ten studies, but not in the other four. It should be noted that only the study by Faure et al. [22] proposed two exercise sessions and two rest sessions. Physical activity was almost always performed on a treadmill or ergocycle (in water in three studies) at an intensity of 60 to 70% of VO_2max_ (or HR_max_ [29]). The mean duration of exercise was 45 ± 10 min. In the study by Ahmed et al. [21], participants performed two 2 h sessions of military activities, whereas that of Kojima et al. [28] included high-intensity interval training based on repeated sprints.

### 3.7. Changes in Energy Intake

The mean change in energy intake during heat exposure relative to control conditions was −635 kJ (CI: −1152 to −118) for all studies combined (*n* = 6) and −541 kJ (CI: −1212 to 130) for studies including physical activities (*n* = 4).

The mean change in energy intake during cold exposure was +750 kJ (CI: 216 to 1283) relative to control conditions across all studies (*n* = 11) and +1051 kJ (CI: 462 to 1639) in studies including physical activities (*n* = 8). A 660 kJ decrease in energy intake was found in one study [21]; this observation conflicts with the increase observed in the majority of cold studies.

### 3.8. Risk of Bias across Studies

**Figure 3:**
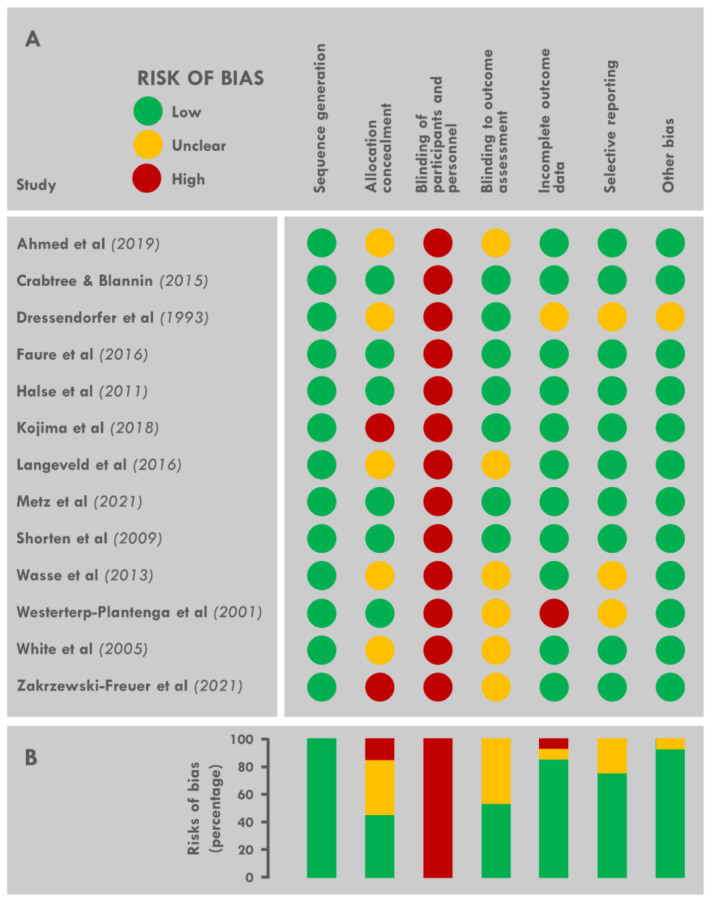
Risk of bias assessment for each randomized controlled trial (**A**) and cumulative analysis for each domain (**B**).

Full details of the risk of bias assessment are provided in Figure 3A. The principal problems detected in the 13 randomized controlled trials were a high risk of bias due to (1) the lack of blinding of participants and study staff to temperature conditions, and (2) the secret assignment of temperature before the start of the study. Information on the blinding methodology for the assessment of energy intake was not clear in most studies. Figure 3B summarizes the proportion of trials with low, unclear, and high risks of bias for each domain. All studies were of high quality according to the Cochrane review criteria.

### 3.9. Meta-Analysis

**Figure 4:**
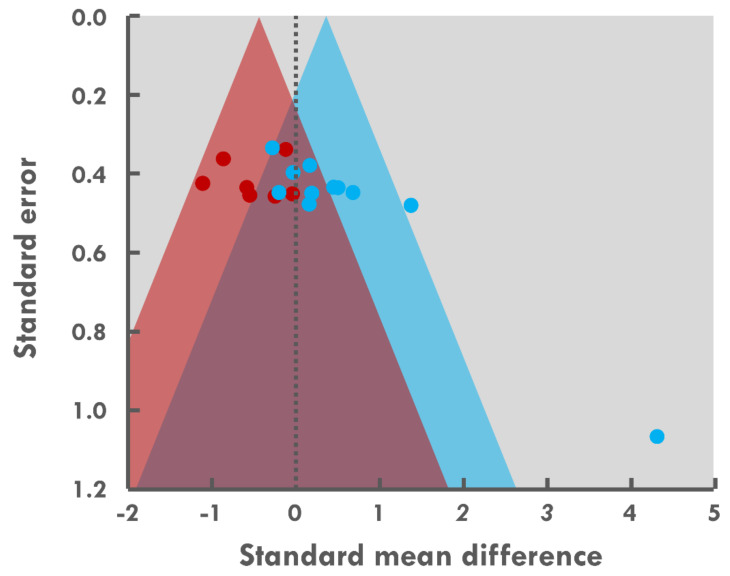
Funnel plot for random-effects model-based meta-analysis of energy intake during or after exposure to heat (red area and dots) or cold (blue area and dots) relative to a thermoneutral environment.

Energy intake in hot conditions was examined in seven studies. Two studies examined this effect without physical activity. Both subgroups were included in the meta-analysis, which identified a small effect of thermal exposure with heat inducing a decrease in energy intake (ES = −0.39, *p* = 0.022, CI: −0.73 to 0.06; Figure 2). The heterogeneity between these studies was low (I^2^ = 2.91%; Q = 4.83 < df = 5, *p* = 0.437). The funnel plot and the Egger statistical test revealed no evidence of publication bias (Figure 4, Egger test *p* = 0.50). The 95% credible interval as −0.76 to −0.03. The two studies that did not include physical activity in their protocol did not fall within this interval [20,22].

An analysis of the studies including physical activity (*n* = 4) revealed only minor shifts in effect size, with no impact on the overall significance of the mean effect (−0.31 *p* = 0.132, CI: −0.71 to 0.09).

Energy intake in cold conditions was examined in 11 studies. Three studies investigated the effects of cold exposure in the absence of physical activity. Both subgroups were included in the meta-analysis, which identified a small effect of temperature with cold exposure leading to an increase in energy intake (ES = 0.44, *p* = 0.019, CI: 0.07 to 0.81; Figure 2). The heterogeneity between these studies was moderate (I^2^ = 48.47%; Q = 25.74 < df = 10, *p* = 0.004), and the actual effect varied between studies. The funnel plot and the Egger statistical test revealed evidence of publication bias (Figure 4, Egger test *p* = 0.001). The 95% credible interval was −0.47 to 1.35. The mean values for the studies by Dressendorfer et al. [38] and Metz et al. [29] did not fall within these intervals.

An analysis of studies including physical activity (*n* = 8) showed an increasing shift in effect size (*p* = 0.042, CI: 0.02 to 1.32), but with high heterogeneity (I^2^ = 76.46 %; Q = 24.60 < df = 7, *p* = 0.001).

## 4. Discussion

In recent decades, the impact of acute exposure to cold or heat on energy intake has been investigated in several studies with contrasting results. The purpose of this meta-analysis was, therefore, to assess the statistical and methodical robustness of the published results. The primary finding was that exposures to cold and heat modify energy intake in an opposite manner. Indeed, exposure to cold tends to increase energy intake, whereas exposure to heat tends to decrease it. Exercise appears to modulate these effects, although this modulation requires confirmation, given the small number of studies available.

As previously established [4], this meta-analysis highlighted the difficulties assessing the effects of ambient temperature on energy intake in humans. Indeed, the considerable variability of the protocols necessarily shuffles undoubtedly affected the changes in energy intake, complicating the observation of a constant effect. Energy intake was measured by the same gold-standard procedure (weighing of the consumed foods) in all the included studies, but the food selected and the timing, number, and nature of the meals (buffet or meals with less than three items) varied considerably between the studies included. Moreover, temperature of the items served may jeopardize the relationships between palatability, thermal comfort, and body temperature potentially affecting intake [40,41,42,43]. However, the range of temperatures was large (cold, warm, and/or room temperature) and often weakly reported. The thermal stress was applied in different manners, with different temperatures and different relative humidifies, differences in the nature of the thermal stress (immersive or aerial), durations of exposure, and in whether or not the thermal stress was maintained during meals. Clothing management was also heterogeneous, with the same outfit imposed in some studies, but a modulation of clothing according to the thermal conditions or a free choice of clothing by the participants in others. Moreover, thermal stress was not systematically objectively assessed by similar measurements of thermal perceptive and physiological markers, making an inter-study comparison difficult. Finally, the presence or absence of exercise may influence appetite responses, making it difficult to determine with certainty the isolated effects of thermal stress on energy intake. Energy intake is an outcome subject to a high degree of interindividual variability [44,45,46]. As thermal stress is also experienced very differently between individuals [47,48,49] and physiological and perceptive markers may be very weakly associated [50,51], we expected to find inconsistent effects of heat and cold exposures on energy intake. However, despite all these limitations, we found that exposures to heat and cold had clear effects, as the changes in energy intake were small-to-modest but appear robust under the circumstances. The effects of heat exposure were considered highly homogeneous, whereas those of cold exposure were not. However, this considerable heterogeneity is probably due largely to the exceptional results of the study by Dressendorfer et al. [38]. Indeed, I^2^ and Q (two indices of heterogeneity) decreased from 48.5 to 18.4% and from 25.7 to 11.3, respectively, upon removal of this study.

The relevance of these changes in energy intake for athletes and military personnel remains to be determined. An increase in energy expenditure or a decrease in energy intake leads to a negative energy balance [52], which may compromise performance and physiological processes if chronic [14,15]. The physiological significance of the changes in energy intake detected in this meta-analysis should, therefore, be analyzed according to changes in energy demand. Relative to temperate conditions, total energy expenditure seems to increase, at most, marginally (5%) [53,54,55] in hot conditions, due to an increase in ventilation and sweat gland activity [53]. However, this slight increase is generally compensated by strategies to avoid heat by moving into the shade or air-conditioned rooms, for example [1]. Exposure to heat is, therefore, likely to modify energy expenditure only slightly. The decrease in energy intake observed in this meta-analysis should, therefore, lead to a negative energy balance. Over a 10 day period, exposure to heat may create an additional energy deficit of 26 MJ (if we consider a diurnal period of 16 h), corresponding to a loss of 895 g of body mass (444 g of fat mass and 452 g of fat-free mass if we consider 68% of body mass loss to be attributable to fat mass [7] and energy equivalents of 18.4 and 39.8 kJ.g^−1^ for protein and fat, respectively [56]). This estimate, although hypothetical, indicates that heat exposure may significantly increase the energy deficit. In conditions similar to those in the sedentary studies included in this meta-analysis (16–22 °C vs. 22–28 °C for 2.5–84 h), the cold-induced increase in energy expenditure is more consistent than that induced during heat exposure [23,30,54], although it nevertheless remains modest (+7%). This increase can probably be explained by “non-shivering thermogenesis” by the brown adipose tissue [57] to regulate central temperature [23]. The increase in resting metabolic rate can reach 250% in cases of extremely cold stimuli triggering high-intensity shivering [30]. The increases in energy demand during exercise in the cold are less pronounced and were inconsistently observed [16,29,58,59] due to exercise-induced internal heat production. In these active conditions, energy expenditure is probably not increased by exposure to cold if the exposure ceases just after exercise. This meta-analysis highlighted an orexigenic effect of acute exposure to cold, but it is unclear whether the small increases in energy intake observed were sufficient to compensate for the increases in energy demand. In the studies (regardless of the presence or absence of physical exercise) in which energy expenditure and intake were both measured [16,17,23,27,29], the mean increase in energy intake was found to be greater (+586 kJ (138 to 1124 kJ)) than the increase in energy expenditure (+144 kJ (−137 to 500 kJ)). These comparisons suggest that individuals exposed to acute moderate cold at rest or during exercise are not at risk of facing a negative energy balance.

Physical exercise generally leads to an energy deficit because the energy expended during the exercise session is only marginally compensated by an increase in energy intake during subsequent ad libitum meals [60]. No compensatory increase in energy intake occurs just after exercise, but there is often a transient decrease in hunger [61] accompanied by a decrease in the plasma concentration of acylated ghrelin (an orexigenic hormone) and an increase in the plasma concentration of anorexigenic hormones (peptide YY (PYY), glucagon-like peptide 1 (GLP-1), and pancreatic polypeptide (PP)) [62,63]. Even if small and short-lived [61], we cannot rule out the possibility that these effects can cancel out those of ambient temperature described above. In this meta-analysis, in studies including physical exercise, this exercise was performed in both hot/cold and thermoneutral conditions, implying that the effects of the exercise on energy intake should have been similar. However, it could be hypothesized that the magnitude of the exercise-induced changes could potentially decrease or cancel out those of thermal exposure. In this meta-analysis, the overall anorectic effect induced by heat exposure slightly decreased with the withdrawal of rest conditions (−0.39 (*p* = 0.022) and −0.31 (*p* = 0.132) with and without rest sessions, respectively). This could be interpreted as indicating that the effect of heat is slightly blunted by physical exercise. Conversely, the withdrawal of sedentary sessions did not weaken the evidence for a cold-induced orexigenic response (0.44 (*p* = 0.019) and 0.67 (*p* = 0.042), with and without the sedentary sessions, respectively). The effect of cold exposure does not, therefore, appear to be modified by exercise. Furthermore, no orexigenic effect of cold was found in an analysis including only the three sedentary sessions (0.15 (*p* = 0.55), results not shown) [20,23,27], suggesting that sedentary acute exposure (<1 day) to cold (between 10 and 18 °C) induces a very weak orexigenic response relative to thermoneutral control conditions. However, there are currently too few published studies to formulate sound hypotheses, and further studies are required to investigate the interactions between thermal exposure and exercise. It remains unclear how exposures to cold and heat modulate energy intake independently of physical exercise.

Several hypotheses explaining these changes in energy intake may be proposed. The first and most obvious is that changes occur in the plasma concentrations of hormones involved in energy homeostasis [64,65]. Gastrointestinal hormones modulate appetite, some increasing it (ghrelin) and other decreasing it (PYY, GLP-1, cholecystokinin (CCK), and PP). Leptin, which is secreted by the adipose tissue, also plays an anorexigenic role. In the studies included in this meta-analysis, changes in the plasma concentrations of these hormones were sometimes assessed alongside energy intake: evidence of a heat-induced increase in plasma leptin [19,66] and PYY [19] concentrations and a decrease in plasma total ghrelin concentration [22] and a cold-induced increase in plasma ghrelin concentration [17,39,66] and a decrease in plasma leptin concentration [39] has been reported. Moreover, support for these cold-induced modifications has been provided by other studies focusing on the hormonal response during thermal stress [67,68]. These changes are consistent with the changes in energy intake, but it has yet to be demonstrated that they actually induced the concomitant changes in energy intake. Indeed, Mandic et al. [66] reported significant negative associations between PYY and GLP-1 fluctuations and energy intake, but not with ghrelin and leptin concentrations, implying that the interactions between peripheral modulators of food intake and actual food intake may actually be more complex. Finally, many studies reported no effect of the various acute thermal stresses on the levels of these hormones [18,20,25,26,28], with some nevertheless observing changes in energy intake [18,20], suggesting a role for other mechanisms acting directly in the central nervous system, including the hypothalamus in particular.

High ambient temperatures have been found to increase the activity of the arcuate nucleus (ARC) in rats [69], but the neurons specifically activated were not determined. A decrease in mRNA levels for neuropeptide Y (NPY) has also been reported in chicks and rats [70,71]. As NPY is a peptide produced by the NPY/AgRP (Agouti-related protein) neurons in the ARC, which stimulates food intake [72], this downregulation of NPY may partly explain the heat-induced anorexigenic response. Cold exposure seems to induce the opposite effect, leading to an increase in NPY concentration at its hypothalamic release sites [73,74], despite a decrease in production in the ARC [75] and a decrease in mRNA levels [76]. It has been suggested that the decrease in the antipyretic effect of NPY to facilitate thermogenesis takes precedence over the change in feeding behavior [77]. AgRP neurons are rapidly activated in mice [78], suggesting that the perception of cold is sufficient to trigger these responses. Notwithstanding the small number of studies published, the available evidence suggests that thermal exposure may modify the activity of the ARC and its production of peptides, consistent with the observed changes in energy intake. Another possible mechanism involves peripheral and central glucose metabolism. During [79] and after exercise [80], and in the postprandial phase [81,82], heat exposure increases plasma glucose levels, mostly due to an increase in hepatic glucose production [83] stimulated by an increase in catecholamine release [84]. A small decrease in glycemia is thought to trigger meal initiation [85], so any increase in plasma glucose levels should delay eating or decrease appetite. A peripheral infusion of glucose was found to decrease food intake in rats [86,87] via vagal afferences to the brainstem and the hypothalamus [88,89], but this effect was not observed in humans [90]. Moreover, several hypothalamic areas (ARC, ventromedial, paraventricular, and lateral hypothalamus) [91,92] contain glucose-excited (GE) or glucose-inhibited (GI) neurons that are triggered by rises and falls in glucose concentration, respectively [93,94]. In the ARC, POMC/CART neurons (considered anorexigenic) were found to be GE neurons [95], whereas NPY/AgRP neurons (considered orexigenic) were found to be GI neurons [95]. Heat-induced hyperglycemia may, therefore, induce an anorexigenic response.

Other hypotheses currently lacking experimental support may also partly explain the changes in energy intake. An impact of the redistribution of blood flow during thermal exposure has been suggested as a possible mechanism [17,20]. Heat exposure diverts some of the blood flow away from the core and the muscles to the skin to facilitate heat dissipation, whereas cold exposure increases core blood flow to limit heat dissipation and maintain core temperature. These adjustments may affect splanchnic blood flow and facilitate or impede the circulation of gut-derived appetite hormones to the brain. Moreover, the decrease in cerebral blood flow during hyperthermia [96] may also reduce the impact of hormones. One recent study [97] reported interactions between skin blood flow, food intake, and ambient temperature. This hypothesis may, therefore, be worthy of further investigation. Moreover, Westerterp-Plantenga et al. [27] demonstrated a positive association between the decrease in rectal temperature when temperature was decreased from 22 °C to 16 °C sessions and the percent increase in energy intake, indicating that overeating during cold exposure may preserve core temperature by increasing diet-induced thermogenesis. Finally changes in food reward value and its underlying components of implicit motivation (wanting) and explicit sensory pleasure (liking) may affect food choices and/or the amount of food consumed, and, therefore, energy intake [98]. The Leeds Food Preference Questionnaire (LFPQ) [99], used to assess food reward, is regularly used after an exercise session [44,100] or training [101], but only occasionally in assessments of the effects of environmental conditions. However, the preference for sweet foods and a liking for high-fat and savory foods were shown to increase during a fifteen day expedition to Greenland [102] and a four day rapid ascent in the Alps [103], respectively. However, the impact of environmental temperature was not assessed and remains unknown. It would, therefore, be of interest to use this questionnaire in laboratory and field contexts to determine whether the changes to energy intake during heat and cold exposures are associated with changes in food reward.

This meta-analysis deals only with the acute effects on energy intake of exposure to cold and heat because no long-term randomized controlled studies are available. This is not surprising because the imposition of a similar multi-day sequence with at least two sets of thermal conditions would be a major constraint. This approach is possible in laboratory conditions, as demonstrated by the performance of a 31 day study simulating the ascension of Everest [104]. It is also feasible in the field and has been used in the military context [2,105]. However, both approaches have intrinsic major flaws: laboratory protocols are costly and the confinement they entail might generate non-negligible detrimental psychological effects altering food intake, and field studies are characterized by many biases (non-controlled ambient temperatures and activities, differences in the foods available, and participants, etc.) and the contextual limitations of food intake are numerous and cumulative and may differ between thermal conditions [7]. Nevertheless, such assessments of long-term exposure remain essential because it is unknown whether the different thermal stresses continue to affect energy intake similarly over time. Just as exercise-induced energy deficits gradually disappear due to an increase in spontaneous energy intake [102,106,107], it is possible that the effects described in this meta-analysis change over time.

## 5. Conclusions

Despite major methodological differences between the studies included, a clear effect of acute heat/cold exposure on energy intake emerged. This meta-analysis highlights a modest orexigenic effect of cold exposure and a small anorexigenic effect of heat exposure. For populations in which it is important to avoid energy deficiency [108,109], such as athletes and military personnel, these observations indicated that training, operations, and life in hot conditions may necessitate attention to an increase in the risk of inadequate energy intake. In cold conditions, this should not be a problem, provided that sufficient food is available [7]. For populations aiming to lose body mass, training or living in hot conditions would appear to be advantageous because the increase in energy demand is not spontaneously compensated by food intake, contrary to what is observed for cold exposure. Further studies may help to identify the temperature threshold for it to affect energy intake or to improve our understanding of the role of relative humidity, particularly in hot conditions, neither of which were explored in the studies included in this meta-analysis.

## Data Availability

The data presented in this study are available on request from the corresponding author.
